# Retracted: Next Generation Sequencing: Potential and Application in Drug Discovery

**DOI:** 10.1155/2020/1757504

**Published:** 2020-08-25

**Authors:** The Scientific World Journal

The Scientific World Journal has retracted the article titled “Next Generation Sequencing: Potential and Application in Drug Discovery” [1]. The article was found to contain a substantial amount of material from the following published article, cited as reference [15]:

“Recent Patents and Advances in the Next-Generation Sequencing Technologies,” by Biaoyang Lin, Jun Wang, and Yin Cheng published in Recent Patents on Biomedical Engineering, 2008, DOI: 10.2174/1874764710801010060 (http://www.eurekaselect.com/95056/article/recent-patents- and-advances-next-generation-sequencing-technologies) [2].
